# Feasibility of extended transforaminal approach (medial subchoroid) for resection of a benign aqueductal tumor in a patient with type 1 neurofibromatosis

**DOI:** 10.3171/2023.1.FOCVID22155

**Published:** 2023-04-01

**Authors:** Jose M. Narro-Donate, Jose J. Guil-Ibañez, Maria José Castelló-Ruiz, Fernando García-Pérez, Gaizka Urreta-Juarez, José Masegosa-González

**Affiliations:** Neurosurgery Department, Hospital Universitario Torrecárdenas, Almería, Spain

**Keywords:** ventricular endoscopy, neuroendoscopy, transforaminal approach

## Abstract

The extended transforaminal endoscopic approach allows visualization and manipulation of the third ventricle posterior structures in a safe and comfortable manner. The medial subchoroidal approach has been described as a feasible alternative to the classical transchoroidal approach.

In this video, the authors present the case of a 14-year-old male with a history of neurofibromatosis type 1 who was referred to our department after presenting with headaches and diplopia for 2 weeks. Suspecting an aqueduct tumor, the authors performed an endoscopic surgical procedure through a single approach with third cisternostomy and resection of the tumor that produced the stenosis.

The video can be found here: https://stream.cadmore.media/r10.3171/2023.1.FOCVID22155

## Transcript

We present the feasibility of an extended transforaminal approach (medial subchoroid) for resection of a benign aqueductal tumor in a patient with type 1 neurofibromatosis. No relevant financial disclosures are declared.

### 0:36 Clinical Presentation.

This is a 14-year-old male with a history of type 1 neurofibromatosis followed by multiple neuromas in the lumbar plexus. He was referred to our department after presenting with headaches and diplopia for 2 weeks. His physical exam was remarkable for a right cranial nerve VI palsy with no other focal symptoms. Funduscopic examination revealed incipient bilateral papilledema. An emergent MRI was performed showing a triventricular hydrocephalus with a suspected lesion in the upper part of the aqueduct of Sylvius.

### 1:10 Preoperative MRI.

The T2-weighted images show dilatation of the ventricular system with associated transependymal edema and signs of endocranial hypertension. The gadolinium-enhanced sagittal T1 sequences show no significant enhancements without clear pathology. Nevertheless, in sagittal CISS sequences, we can observe a normal-sized fourth ventricle with the suspicion of a lesion in the most cranial part of the aqueduct marked by the red arrow.

### 1:42 Preoperative Planning.

The surgical planning. We use general anesthesia with the patient in a supine position. The head is slightly elevated (about 20°) with a right frontal arciform incision. No Mayfield clamp is used, and electromagnetic navigation is preferred [StealthStation S8 surgical navigation system, Medtronic]. For the extended transforaminal approach, it is important to measure the size of the foramen of Monro because it is a marker of the difficulty we will encounter during the procedure.^1^ Because we want to add a ventriculostomy during the surgery, we plan a trajectory that is the bisector of both trajectories to both desired targets with a single burr hole in the precoronal position. A rigid endoscope was used with a 6° view angle [LOTTA, Karl Storz].

### 2:26 Trajectory.

The choice of trajectory is shown in this picture. The two ideal trajectories would require two different burr holes, but the extended transforaminal approach allows us to simplify the surgery by performing only one burr hole.^2–4^ The measures of foramen of Monro are shown in this image. In this case, the trephine was made 1.7 cm anterior to the coronal suture and 2.8 cm from the midline as shown in this 3D CT reconstruction.

### 3:01 Surgery.

Once we enter the ventricle, we perform a careful inspection of the structures we encounter. We can see a dilated foramen of Monro showing the third ventricle floor structures. We identify structures that let us confirm that we are in the right lateral ventricle, such as the septal vein, the choroid plexus, and the thalamostriate vein.

In this case, the confluence of the septal and the thalamostriate vein allows us to extend the foramen lateral to the choroid plexus and medial to the thalamostriate vein, in a medial subchoroid fashion, thus reaching the posterior third of the third ventricle.^5^

### 3:41 Expanded Transforaminal Approach.

With gentle and delicate maneuvers, we can dissect the tenia choroidea in a safe manner, avoiding large tractions on the vascular nerve structures and reaching the posterior part of the third ventricle. A blunt dissection is performed by the endoscope itself without no need to coagulate veins. At this point, we can identify the aqueduct of Sylvius and a tumor in the upper part that clearly obstructs the cerebrospinal fluid circulation.

### 4:15 Surgery.

Surgical excision of the lesion is performed with standard tools.

### 5:01 Tumoral Final Resection.

Once the lesion is resected, patency of the aqueduct is observed with free circulation of cerebrospinal fluid through it. No complications secondary to resection were observed.

### 5:13 Ventriculostomy.

At this point, we decided to add a safety ventriculostomy by guiding the endoscope to the anterior part of the floor of the third ventricle. Mammillary bodies and premammillary membrane are identified. With the bipolar tool, we perforate the membrane and then use a 4-Fr Fogarty to dilate the perforation. Once completed, the endoscope is introduced to confirm the correct perforation of the Liliequist membrane. The basilar artery is identified with no significant surgical complications.

### 5:57 Damage Verification.

When the endoscope is removed, the absence of lesions on the fornix is confirmed.^6^ The medial subchoroid approach is shown without damage to the venous structures. Intraoperative reduction of the ventricular size is observed.

### 6:30 Clinical Outcome.

Clinical outcome and follow-up. The postoperative showed no complications, and the patient was discharged 72 hours after surgery. Evident improvement of the headache was observed and the sixth nerve palsy resolved 1 month after surgery. Follow-ups at 6, 12, and 24 months confirmed excellent radiological and clinical evolution with no signs of tumor recurrence.

### 6:56 Follow-Up.

After 2 years, the T2-weighted imaging sequence shows complete resolution of the hydrocephalus. In the sagittal plane, it is possible to confirm the patency of the ventriculostomy and the associated flow artifact that can also be seen in the aqueduct. Thank you.

## Disclosures

The authors report no conflict of interest concerning the materials or methods used in this study or the findings specified in this publication.

## Author Contributions

Primary surgeon: Narro-Donate. Editing and drafting the video and abstract: Narro-Donate. Critically revising the work: Urreta-Juarez, Castelló-Ruiz, Masegosa-González. Reviewed submitted version of the work: Narro-Donate, Guil-Ibañez, Castelló-Ruiz, García-Pérez, Urreta-Juarez. Approved the final version of the work on behalf of all authors: Narro-Donate. Supervision: Masegosa-González. Video narration: Narro-Donate.

## Supplemental Information

### Patient Informed Consent

The necessary patient informed consent was obtained in this study.
